# Supplementary material for: Heat adaptation of phage T7 under an extended genetic code
Source: Virus Evol. 2021 Dec 1;7(2):veab100. doi: 10.1093/ve/veab100 (PMC8923235; doi:10.1093/ve/veab100)
Supplement: veab100_Supp [file veab100_supp.zip › Sequencing_Analysis.zip!/Sequencing_Analysis/S3/marginal.html]

BRESEQ :: Marginal Predictions


|  |  |
| --- | --- |
|  | ***breseq***  version 0.30.1    mutation predictions \| marginal predictions \| summary statistics \| genome diff \| command line log |

| Marginal read alignment evidence | | | | | | | | | | | |
| --- | --- | --- | --- | --- | --- | --- | --- | --- | --- | --- | --- |
|  | seq id | position | | ref | new | freq | score (cons/poly) | reads | annotation | genes | product |
| \* | NC\_001604.1 | 29,691 | 0 | A | G | 63.9% | 1272.5 / inf | 1475 | intergenic (–/–) | *–/–* | –/– |
| \* | NC\_001604.1 | 26,306 | 0 | A | G | 62.2% | 1388.1 / inf | 1812 | intergenic (–/–) | *–/–* | –/– |
| \* | NC\_001604.1 | 34,973 | 0 | C | T | 49.2% | 120.2 / inf | 1662 | intergenic (–/–) | *–/–* | –/– |
| \* | NC\_001604.1 | 35,254 | 0 | A | G | 45.9% | 370.5 / inf | 1700 | intergenic (–/–) | *–/–* | –/– |
| \* | NC\_001604.1 | 28,691 | 0 | A | C | 44.5% | 97.1 / inf | 1448 | intergenic (–/–) | *–/–* | –/– |
| \* | NC\_001604.1 | 34,944 | 0 | G | A | 39.0% | 1111.0 / inf | 1637 | intergenic (–/–) | *–/–* | –/– |
| \* | NC\_001604.1 | 28,692 | 0 | A | G | 36.2% | 1098.1 / inf | 1448 | intergenic (–/–) | *–/–* | –/– |
| \* | NC\_001604.1 | 35,366 | 0 | C | A | 32.8% | 2351.2 / inf | 1848 | intergenic (–/–) | *–/–* | –/– |
| \* | NC\_001604.1 | 14,351 | 0 | A | T | 28.0% | 2607.8 / inf | 1779 | intergenic (–/–) | *–/–* | –/– |
| \* | NC\_001604.1 | 14,348 | 0 | T | C | 27.7% | 2286.8 / inf | 1813 | intergenic (–/–) | *–/–* | –/– |
| \* | NC\_001604.1 | 19,319 | 0 | T | C | 23.2% | 2436.2 / inf | 1547 | intergenic (–/–) | *–/–* | –/– |

| Marginal new junction evidence (sorted from low to high skew) | | | | | | | | | | | |
| --- | --- | --- | --- | --- | --- | --- | --- | --- | --- | --- | --- |
|  | | seq id | position | reads (cov) | reads (cov) | score | skew | freq | annotation | gene | product |
| \* | ? | NC\_001604.1 | = 16508 | 832 (0.480) | 277 (0.160) | 147/586 | 6.7 | 27.7% | intergenic (–/–) | *–/–* | –/– |
|  | ? | NC\_001604.1 | 16970 = | 637 (0.380) |  |  |  |  | intergenic (–/–) | *–/–* | –/– |
| \* | ? | NC\_001604.1 | = 19635 | 1486 (0.860) | 128 (0.080) | 91/590 | 14.0 | 8.1% | intergenic (–/–) | *–/–* | –/– |
|  | ? | NC\_001604.1 | 19645 = | 1460 (0.860) |  |  |  |  | intergenic (–/–) | *–/–* | –/– |
| \* | ? | NC\_001604.1 | = 19585 | 1527 (0.890) | 42 (0.020) | 37/586 | 27.2 | 2.7% | intergenic (–/–) | *–/–* | –/– |
|  | ? | NC\_001604.1 | 19598 = | 1506 (0.900) |  |  |  |  | intergenic (–/–) | *–/–* | –/– |
| \* | ? | NC\_001604.1 | = 19664 | 1662 (0.970) | 33 (0.020) | 26/592 | 31.9 | 2.0% | intergenic (–/–) | *–/–* | –/– |
|  | ? | NC\_001604.1 | 19680 = | 1629 (0.960) |  |  |  |  | intergenic (–/–) | *–/–* | –/– |
| \* | ? | NC\_001604.1 | = 281 | 1619 (1.060) | 375 (0.230) +TAAAGACCAGACC | 16/508 | 34.0 | 17.7% | intergenic (–/–) | *–/–* | –/– |
|  | ? | NC\_001604.1 | 282 = | 1822 (1.060) |  |  |  |  | intergenic (–/–) | *–/–* | –/– |
| \* | ? | NC\_001604.1 | = 7972 | 2034 (1.180) | 21 (0.010) | 20/588 | 34.7 | 1.1% | intergenic (–/–) | *–/–* | –/– |
|  | ? | NC\_001604.1 | 8524 = | 1958 (1.160) |  |  |  |  | intergenic (–/–) | *–/–* | –/– |
| \* | ? | NC\_001604.1 | 295 = | 2222 (1.290) | 18 (0.010) | 13/560 | 37.9 | 0.86% | intergenic (–/–) | *–/–* | –/– |
|  | ? | NC\_001604.1 | = 281 | 2083 (1.300) |  |  |  |  | intergenic (–/–) | *–/–* | –/– |
| \* | ? | NC\_001604.1 | = 13586 | 2 (0.000) | 28 (0.020) | 14/592 | 38.4 | 65.1% | intergenic (–/–) | *–/–* | –/– |
|  | ? | NC\_001604.1 | 14356 = | 28 (0.020) |  |  |  |  | intergenic (–/–) | *–/–* | –/– |
| \* | ? | NC\_001604.1 | = 8335 | 1786 (1.040) | 7 (0.000) | 6/584 | 44.4 | 0.39% | intergenic (–/–) | *–/–* | –/– |
|  | ? | NC\_001604.1 | 8939 = | 1866 (1.110) |  |  |  |  | intergenic (–/–) | *–/–* | –/– |
| \* | ? | NC\_001604.1 | 8888 = | 2048 (1.190) | 3 (0.000) | 3/592 | 47.8 | 0.15% | intergenic (–/–) | *–/–* | –/– |
|  | ? | NC\_001604.1 | 8897 = | 2013 (1.190) |  |  |  |  | intergenic (–/–) | *–/–* | –/– |
